# Do Wealth Shocks Affect Health? New Evidence from the Housing Boom

**DOI:** 10.1002/hec.3431

**Published:** 2016-11-09

**Authors:** Eleonora Fichera, John Gathergood

**Affiliations:** ^1^ Manchester Centre for Health EconomicsDivision of Population Health, Health Services Research & Primary Care; ^2^ School of EconomicsUniversity of Nottingham

**Keywords:** health, wealth, housing wealth, house prices

## Abstract

We exploit large exogenous changes in housing wealth to examine the impact of wealth gains and losses on individual health. In UK household, panel data house price increases, which endow owners with greater wealth, lower the likelihood of home owners exhibiting a range of non‐chronic health conditions and improve their self‐assessed health with no effect on their psychological health. These effects are not transitory and persist over a 10‐year period. Using a range of fixed effects models, we provide robust evidence that these results are not biased by reverse causality or omitted factors. For owners' wealth gains affect labour supply and leisure choices indicating that house price increases allow individuals to reduce intensity of work with commensurate health benefits. © 2016 The Authors. *Health Economics* Published by John Wiley & Sons, Ltd.

## 1 Introduction

In this paper, we use the large exogenous changes in household wealth held across the population caused by the UK house price booms and busts of the last two decades to explore the central question in the economics of health: the impact of wealth gains and losses on individual health. Using individual level survey data for a representative sample of UK home owners, we show that house prices have economically important and statistically significant effects on health.

Wealth gains from house price increases lead reductions for home owners (who are the majority of the population) in the number of medical ailments suffered and lead to improved self‐assessed health. Using a combination of econometric strategies, we show that our estimates are not driven by unobserved shocks at the local level which might correlate with house prices and health. We also show that our results are not attributable to reverse causality or a purely psychological effect whereby changes in prices make households ‘feel better’ psychologically. Our results imply that housing market activity has economically important effects on health for home owners.

Understanding the causal links between economic resources and health (which is assumed to be a normal good as in Grossman, 1972) presents challenges to the researcher (Deaton and Paxson, 1999; Marmot and Bobak, 2000). A reverse causality may also exist (Smith, 1999) or the positive relationship might be explained by additional factors such as family background or genetics (Currie and Stabile, 2003; Dehejia and Muney, 2004; van den Berg *et al.,* 2006).

Previous studies have used exogenous changes in economic resources such as lottery wins, inheritances or estimated income shocks to measure the causal relationship between wealth and health (Lindahl, 2005; Gardner and Oswald, 2007; Michaud and van Soest (2008); Adda *et al.,* 2009; Apouey and Clark, 2011; Kim and Ruhm, 2012; Böckerman *et al.,* 2007). Other studies exploit changes in public policy as a source of exogenous variation in income or wealth, as in Case (2004); Snyder and Evans (2006) and Frijters *et al.* (2005).

Our approach to estimating the effect of wealth on health exploits exogenous variation in the largest single asset for the majority of households: variation in housing wealth due to house price movements. Housing is widely held in the population – the home ownership rate in the UK is approximately 64% (ONS, 2013). Banks *et al.* (2003) estimate that housing wealth accounts for 60% of household financial wealth among households in the UK. A major contribution of our approach is that we exploit a very prevalent form of wealth, and therefore, effects on health are more generalisable to the population.

We estimate the causal effect of house prices on health on the assumption that the geographic variation in the strength and timing of house price movements is conditionally exogenous to individual health. Recently, a large literature has exploited this approach to show that house price movements are important for a range of household activity including household consumption and saving (Campbell and Cocco, 2007; Disney *et al.,* 2010; Attanasio *et al.,* 2009), indebtedness (Hurst and Stafford, 2004), educational choices (Lovenheim, 2011; Lovenheim and Reynolds, 2013) rates of childbirth (Lovenheim and Mumford, 2013; Dettling and Kearney, 2014), demand for long‐term care insurance (Davidoff, 2010) and divorce (Farnham *et al.,* 2011).

We find economically important effects of house prices on individual health. A one standard deviation increase in house prices causes on average a decrease in the number of health conditions suffered by an individual of 6.6% of a standard deviation and an improvement in self‐assessed health (SAH) of approximately 4.3% of a standard deviation. House prices have no effect on the likelihood an individual suffers from depression, anxiety or a stress related illness, or on upon their psychological health as measured by the 12‐point General Health Questionnaire (GHQ‐12). When we estimate models for specific categories of health conditions, we find a broad range of physical and medical conditions that are responsive to house price gains and losses apart from, as expected, chronic conditions.

Our results also show the effects of house prices on health are not purely transitory. We estimate a series of long‐difference specifications exploiting 10‐year differences in our panel data. The size of the estimated effects in these long‐difference specifications is similar to that found in the panel estimates. Further estimates show little evidence for non‐linearity in the relationship between house prices and health. We find evidence that individual health is more responsive to house price gains compared with losses.

The focus of our paper is not on the mechanisms or channels by which wealth gains due to house price movements that affect individual health, but we do find evidence that house prices affect labour supply and leisure choices. One possible explanation for results, therefore, is that wealth gains from house price increases allow individuals to reduce intensity of work with commensurate health benefits. This is consistent with the previous literature which finds positive housing wealth effects on consumption (Campbell and Cocco, 2007).

## 2 Data

**Figure 1:**
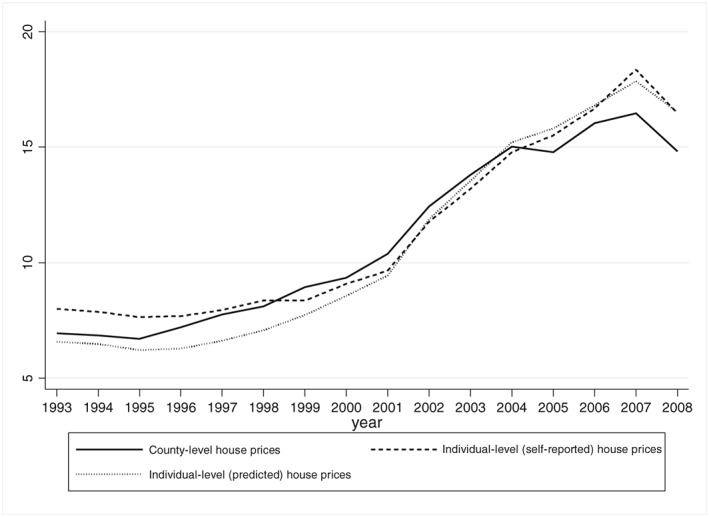
UK aggregate house prices 1993–2008 calculated from sales data values and British Household Panel Survey individual self‐reported data values (all series at year 2000 prices, £0 000s)

**Figure 2:**
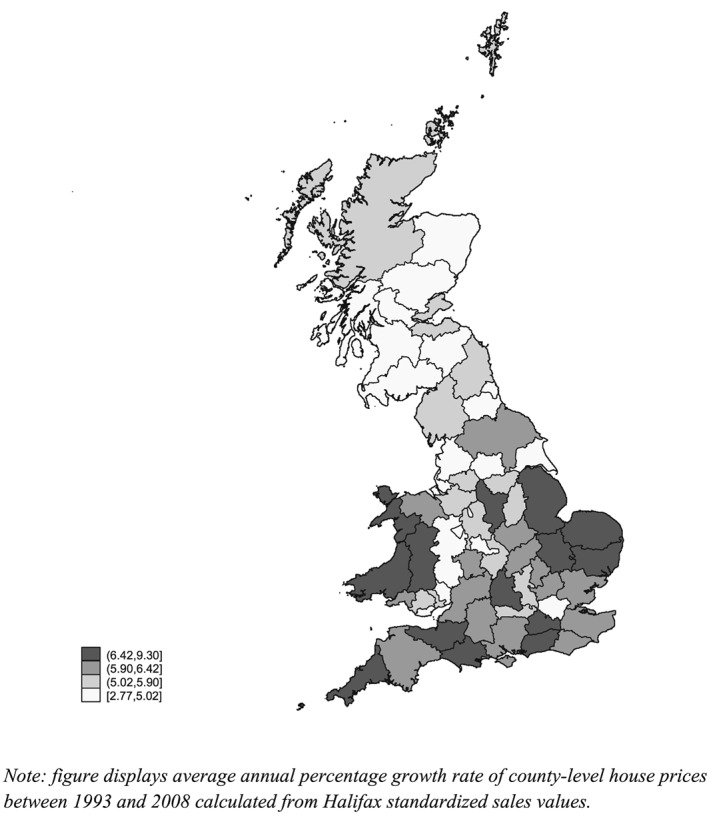
Map of UK county level average annualised real house price growth 1993–2008 calculated from sales data

Figure 1 illustrates UK aggregate real house prices for our sample period 1993–2008. Average house prices as measured from our micro data panel matches the aggregate series closely over the period in its level and dynamics. The faint broken line illustrates predicted prices from an estimated house price model which we later use in econometric analysis. A geographic breakdown of house price growth over the period is shown in Figure 2. As can be seen on the figure, there is considerable geographic variation in house price growth with stronger growth in prices in the south of the UK and weaker growth in the north.

The micro data we use for our analysis is the 1993–2008 British Household Panel Survey (BHPS) with financial variables adjusted to year 2000 prices. Our sample comprises individuals in home owning households aged over 18 who are either the household head or their spouse/partner (our sample begins in 1993 as the question on house value was not included before this wave).

Table 1 provides summary statistics for our sample. As we focus on home owners only, average age in our sample is higher than that in the population. The majority of individuals are aged 49 or over, over 80% are married and 65% are in the labour force. In our sample, the average self‐reported house value (at 2000 prices) is a little below £120 000 and the standard deviation is large at a little below £115 000 reflecting the substantial time series variation in real house prices and variation across counties shown in Figures 1 and 2.

**Table 1:** Summary statistics for 1993–2008 BHPS homeowner sample

	Mean	Standard deviation
Health outcomes		
Number of health conditions (0–13)	1.18	1.30
Self‐assessed health (5 = very poor, 1 = excellent)	2.15	0.87
Suffers depression dummy (1/0)	0.13	0.39
General Heath Questionnaire Score (0–12)	1.40	3.38
Demographics		
Age 16–36	0.19	0.39
Age 37–48	0.27	0.45
Age 49–62	0.28	0.45
Age over 62	0.26	0.44
Marital status dummies		
Married	0.81	0.39
Divorced	0.06	0.23
Widowed	0.07	0.26
Educational qualification dummies		
Degree	0.14	0.35
College	0.17	0.38
High School	0.29	0.45
Employment status dummies		
Employed	0.55	0.50
Self‐employed	0.09	0.28
Unemployed	0.01	0.12
Children (by age) dummies		
Children 0–3	0.06	0.25
Children 4–5	0.18	0.39
Children 6–12	0.13	0.33
Children 13–16	0.04	0.19
Income and house value		
Self‐reported house value	£117 658	£113 112
Gross household income	£26 400	£19 300
County unemployment rate	4.17	2.52
Number of individual‐year observations	105 170	—

BHPS, British Household Panel Survey.

We focus on four health measures. The first measure is the number of current health problems reported by the individual. Focusing on the 13 conditions in our data through the whole sample period, on average individuals identify 1.18 (standard deviation 1.30) conditions with a minimum of zero and maximum of 11. Following Apouey and Clark (2011), we also examine categories of individual health condition outcomes.

Second, the BHPS includes a question relating to SAH in all waves except 1999. The question is ‘compared to people of your own age, would you say your health over the last 12 months on the whole has been: excellent, good, fair, poor or very poor?’ SAH is a commonly used measure of health status, although there is some evidence SAH measures are unreliable and vulnerable to priming (Crossley and Kennedy, 2002). The mean SAH score on a scale of 1 = excellent to 5 = very poor is 2.15; hence, the average respondent's SAH is a little worse than ‘good’.

Our third and fourth health measures focus specifically on psychological health. Our third measure is whether the individual reports they are currently suffering ‘anxiety, depression or bad nerves’ in response to the interviewer showcard prompt. Thirteen per cent of individual‐year observations are for a ‘yes’ answer to this question.

The fourth measure is the GHQ‐12, which is the 12‐question instrument used by psychological health primary assessors to gauge indications of psychological distress (typically family doctors or psychiatric nurses). The BHPS includes the full 12‐question instrument, and this has been used in previous studies of wealth effects on psychological health (Apouey and Clark, 2011; Gathergood, 2012). We code the GHQ‐12 to a value of 12 for poorest psychological health and 0 for best psychological health. The mean score is 1.4 indicating that the average respondent identifies a little less than one and a half psychological health behaviours associated with stress and anxiety among the 12 behaviours.

## 3 Methodology

### 3.1 Main models

We estimate the following baseline econometric model for home owners in the BHPS sample from 1993 to 2008:

(1) $$\text{healt}h_{ict}=\alpha +\beta_{1}\ln{\left(hp\right)}_{ict}+\beta_{2}U_{ct}+\beta_{3}X_{ict}+\varphi_{i}+\vartheta_{ct}+\varepsilon_{ict}$$

where *health* denotes a dependent variable measuring the individual's health status (in our models, these variables are the number of health conditions, SAH, the depression dummy variable and GHQ score), *i* denotes an individual, *φ*_*i*_ is the unobserved time‐invariant individual effect, *c* denotes county of residence and *t* denotes year. The variable *ln(hp)* is the natural log of the self‐reported house price, *U* is the local unemployment rate and *X* is a set of socio‐economic characteristics and control variables. Equation (1) is estimated with linear probability models (LPM) with within‐group fixed effects (FE). There is a substantial geographic component to house prices in our data, and hence, it is likely that errors are correlated spatially. Therefore, we cluster our standard errors at the county level (and separately at the regional level). We find similar results when clustering standard errors at both levels of geographic definition.

Our main interest is in the variable *health* and the coefficient *β_1_* which show how house prices affect health outcomes. The model described in Equation 1 assumes house prices are (conditionally) exogenous to an individual's health. Conditional on the control variables within the model, the estimated coefficient *β_1_* captures the effect of house prices on health, and apart from this, house prices and health should be uncorrelated. This may not be the case if either house prices correlate with geographic factors affecting health, such as the quality of local health provision or local economic conditions, or if there is reverse causality from individual health to house prices.

We cannot directly control for all geographic factors which correlate with house prices. The model includes the county level unemployment rate as a control variable for local labour market conditions which might affect health through work intensity and work related behaviours. Previous studies find local labour market conditions are important for individual health (Ruhm, 2000). To address the possible role for unobserved local factors as omitted variables in Equation 1, we adopt the following approaches.

We include county‐by‐time FE in the model, also used in Lovenheim and Mumford (2013) who study the effects of house prices on fertility choices. With the addition of county‐by‐time FE, the model controls for all unobserved factors common within county and year. In this specification, the coefficient *β_1_* is identified off house price differences among home owners within a county and year. Variation in unobserved spatial factors affecting health is likely to be much less within each year in a county compared with across counties over time.

We then examine the specific health conditions that might have been affected by house prices and re‐estimate Equation (1) with LPM FE models where *health*_*ict*_ indicates each of the 13 conditions in our data. In order to investigate potential long‐run effects, we modify Equation (1) to estimate a long difference model:

(2) $$\tilde{\text{healt}h_{ict}}=\alpha +\beta_{1}\tilde{\ln{\left(hp\right)}_{ict}}+\beta_{2}{\tilde{U}}_{ct}+\beta_{3}{\tilde{X}}_{ict}+{\tilde{\vartheta }}_{ct}+{\tilde{\varepsilon }}_{ict}$$

where the dependent variable is the 10‐year change in the health outcome and the independent variables also enter as 10‐year changes. Individuals enter into our 10‐year differences sample if they are present in the BHPS sample in any year 1993–1998 and are also present in the 10th year following that year. However, Because of the relatively large size of the BHPS sample and low attrition rate, we obtain a sample of approximately 20 000 10‐year differences. Models are estimated as ‘first‐difference’ LPM as a result of which the individual unobserved time‐invariant component is differenced out.

We also estimate Equation (2) for the effects of house prices on mortality. We have limited data on deaths among our survey respondents. We construct data on mortality using the following approach: our information set is limited to recorded deaths where the death of a respondent is identified by another surviving member of the household who remains in the survey in the 11th year. We focus only on deaths between the 10th and 11th year so that we have a set of individual level covariates at year 10. We use the 10‐year changes specification in which the dependent variable is a dummy variable which takes a value of 1 if the respondent is reported as being deceased between the 10th and 11th year. We construct the ‘death’ variable to be equal to one at the last recorded interview in year 10, therefore implying that death has occurred at the end of the BHPS interview year before wave 11. This is a necessary assumption as we do not know the actual date of death. If an individual lives alone and is deceased at the time of the 11th year, their absence from the sample will not be recorded as a death (and there will be no data provided for that individual in that wave of the survey). Finally, we investigate potential mechanisms by estimating Equation (1) on a set of labour market and leisure activities with FE LPM.

### 3.2 Robustness checks

Our main models include variables at different level of aggregation varying either at the individual or county‐level. Although we do not structurally adjust standard errors, we re‐estimate Equation (1) using bootstrapped standard errors. These models rely on the assumption that the sample is representative of the UK population as it is in the BHPS.

Reverse causality presents an alternative possible violation of our assumption that house prices are conditionally exogenous to health. Two forms of reverse causality might exist. First, an individual's psychological health might make them ‘feel’ their house is worth more, or less. To control for this, we also estimate models in which self‐reported prices are replaced with predicted prices:

(3) $$\text{healt}h_{ict}=\alpha +\beta_{1}\hat{\ln{\left(hp\right)}_{ict}}+\beta_{2}U_{ct}+\beta_{3}X_{ict}+\varphi_{i}+\vartheta_{ct}+\varepsilon_{ict}$$

where the first stage regression of house prices is estimated including a vector *z*_*ict*_ = [*π*_*ct*_, *ψ*_*ict*_] of county house prices (*π*_*ct*_) and a broad set of covariates that vary both at the individual and county level and capture characteristics of the house and its features (*ψ*_*ict*_).

We also use a linear FE generalised method of moments (GMM) estimator to deal with the strict exogeneity assumption of the FE estimator (Arellano, 2003). As the GMM can be more efficient in presence of either heteroscedasticity or serial correlation, it has also been used to estimate dynamic wage equations and labour demand (Van Reenen, 1996; Blundell and Bond, 1998). In our application, we estimate Equation (3) whereas vector *z*_*ict*_ contains lags of house prices (ln(*hp*)_*ic*(*t* − *k*)_) of order three and beyond. The GMM estimator ${\hat{\beta }}_{GMM}$ is one that minimises the criterion: $ℶ\left({\hat{\beta }}_{GMM}\right)=N\bar{g}{\left({\hat{\beta }}_{GMM}\right)}^{'}W\bar{g}\left({\hat{\beta }}_{GMM}\right)$. The weighting matrix is such that $\bar{g}\left({\hat{\beta }}_{GMM}\right)$ is as close as possible to zero.

A second cause of reverse causality is adjustments to housing caused by health changes. For example, individuals who experience health improvements which allow them to re‐enter employment may then buy more expensive houses. If such activity occurs, it is likely to occur only with some time lag between the health improvement and change in housing position. These would be shown by the falsification specification where we estimate

(4) $$\text{healt}h_{ict}=\alpha +\beta_{1}\ln{\left(hp\right)}_{ict}+\beta_{2}\ln{\left(hp\right)}_{ic\left(t-k\right)}+\beta_{3}U_{ct}+\beta_{4}X_{ict}+\varphi_{i}+\vartheta_{ct}+\varepsilon_{ict}$$

with lagged values of house prices. We abstract in this specification from the bias that might occur in panels with a small *T* compared to *N* (Nickell, 1981). However, we also estimate our model on the sub‐sample of home owners which excludes those who move home.

The baseline estimates in Equation (1) use LPM. However, the dependent variables include count variables (number of conditions, GHQ‐12 score), categorical responses (self‐assessed health) and a dummy (depression). Therefore, we have also estimated FE Poisson models for the number of conditions and GHQ‐12, a Mundlak correlated random effects effects ordered probit model for self‐assessed health, and a Mundlak correlated random effects effects probit model for the depression dependent variable. Additionally, we model SAH and GHQ as binary variables with SAH that equals to one if SAH is fair, poor or very poor and 0 otherwise, and GHQ equals to one if GHQ is equal to or greater than one. In order to show that our estimates are unaffected by the different assumptions on the unobserved individual component, we also estimate Mundlak linear correlated random effect models.

Equation 1 assumes a linear relationship between house prices and health. We also estimate models in which the house price enters as a polynomial to examine non‐linear effects.

In order to test for differential health responses to house price rises and falls, we partition the house prices vector in Equation (1) in two components:

(5) $$\text{healt}h_{ict}=\alpha +\beta_{1}\ln{\left(hp\right)}_{ict}^{0}+\beta_{2}\ln{\left(hp\right)}_{ict}^{1}+\beta_{3}U_{ct}+\beta_{4}X_{ict}+\varphi_{i}+\vartheta_{ct}+\varepsilon_{ict}$$

House price booms ( $\ln{\left(hp\right)}_{ict}^{0}$) indicate positive house prices deviations from the sample mean, and house price busts ( $\ln{\left(hp\right)}_{ict}^{1}$) indicate negative deviations from the sample mean. This specification allows us to directly test for symmetry in the same model (i.e. *β*_1_ = − *β*_2_).

## 4 Results

### 4.1 Baseline results and robustness checks

Results from estimation of Equation 1 are shown in Table 2. Panel A includes individual level labour market status controls (dummy variables for employed, unemployed and self‐employed with the reference group other labour market states). Panel B excludes these controls, as discussed previously.

**Table 2:** Fixed effects LPM estimates of effect of house prices on homeowner health, (including and excluding individual‐level labour market status control variables)

Panel A: including individual‐level labour market controls				
	(1)	(2)	(3)	(4)
	No. conditions	SAH	Depression	GHQ
House prices	−0.0819** (0.0138)	−0.0377** (0.0101)	−0.00491 (0.00456)	−0.00313 (0.0498)
County unemployment	0.00136 (0.0185)	0.0142 (0.0105)	0.00219 (0.00651)	0.0363 (0.0411)
Annual income	−0.00594 (0.00802)	−0.00562 (0.00597)	0.000464 (0.00231)	0.0228 (0.0260)
Employed = 1	−0.100** (0.0158)	−0.112** (0.00904)	−0.0121** (0.00350)	−0.351** (0.0488)
Self‐employed = 1	−0.123** (0.0248)	−0.136** (0.0166)	−0.00957 (0.00594)	−0.317** (0.0657)
Unemployed = 1	−0.0770* (0.0324)	−0.0715** (0.0212)	0.00599 (0.00888)	0.834** (0.103)
R‐squared	0.077	0.028	0.013	0.020
No. obs.	105 170	97 177	104 992	101 325
No. groups	12 393	12 107	12 384	12 090
No. clusters	64	64	64	64
Panel B: excluding individual‐level labour market controls				
	(5)	(6)	(7)	(8)
	No. conditions	SAH	Depression	GHQ
House prices	−0.0835** (0.0139)	−0.0396** (0.0103)	−0.00491 (0.00460)	−0.00355 (0.0499)
County unemployment	0.00246 (0.0183)	0.0153 (0.0105)	0.00224 (0.00652)	0.0371 (0.0404)
R‐squared	0.075	0.026	0.013	0.017
No. obs.	105 170	97 177	104 992	101 325
No. groups	12 393	12 107	12 384	12 090
No. clusters	64	64	64	64

Standard errors are in parentheses. Years 1993–2008 British Household Panel Survey homeowners sample comprises of head or household and partner/spouse. Models with county are presented by year dummies. Additional covariates included in model not shown in table: age (in age brackets), relationship status dummies, educational achievement dummies and household composition dummies. Cluster standard errors are in parentheses.

LPM, linear probability models; SAH, Self‐assessed health; GHQ, General Health Questionnaire.

* *p* < 0.05.

** *p* < 0.01.

Results in Panel A show house prices reduce the number of health conditions and improve SAH, but have no statistically significant effect upon psychological health. In the first column, the coefficient on the number of health conditions is negative, statistically significant at the 1% level and takes a value of 0.0819. This implies a 100% increase in house prices (which is the standard deviation of house prices in our data) causes a 0.0819 reduction in the number of health conditions, which is approximately 6.5% of a standard deviation. The coefficient on the house price value in the model for self‐assessed of −0.0377 implies a one standard deviation increase in house prices which improves SAH by approximately 4.2% of a standard deviation.

Coefficient estimates for the house price variable in models in which the dependent variable is depression (Column 3), and the GHQ score (Column 4) are both statistically not significant at the 5% level. This result suggests that reverse causality from psychological health causing individuals to ‘feel’ that their house is worth more is not at work in our models, although we return to this issue later.

We present a number of robustness tests of our baseline econometric specification in the accompanying online appendix. We show that the pattern of results is unchanged when we bootstrap standard errors (Table A‐I, Panel A) or include county time‐trends in the model (Table A‐I, Panel B). In both cases, the coefficient magnitudes are near‐identical to those in Table 2.

As house prices are self‐reported, a form of measurement error correlated with health might affect them. To the extent to which this error is time‐variant, it is not accounted for by our main FE specifications. By estimating Equation (3), we show that predicted house prices and house prices instrumented by the vector of lagged values have a strong and statistically significant effect on the number of health conditions (Table A‐II Panel A and B). This coefficient is more than double the one in our main specification. Although SAH is not statistically significant, the size of its coefficient is similar to the main specification.

These results might be due to the smaller sample size or might suggest measurement error to be important for the psychological component of SAH. The falsification test for reverse causality estimated in Equation (4) and reported in Table A‐III shows there are no additional statistically significant effects of lagged house prices on individual health. However, we are cautious in over‐interpreting these results because of the potential Nickell bias.

We have undertaken further robustness tests in which we exclude individuals who move house (Table A‐IV, Panel A) and restrict our sample to a balanced panel (Table A‐IV, Panel B). Less than 5% of our individual‐year observations are for individuals who move home, and when we exclude these households from the estimation sample, we find results are unchanged.

Results from non‐linear models show the same qualitative pattern as the LPM (Table A‐V). The coefficient on house prices in the model for the number of conditions is only one quarter the value of the baseline specification, although the coefficient on house prices in the model for SAH is larger in absolute terms. We also estimate models with Mundlak correlated random effects which reveal the same qualitative pattern of results, although coefficient magnitudes reduce by approximately one quarter. These results are available from the authors on request.

### 4.2 Results for specific health conditions

Results for our general health measures show house prices affect physical health but not psychological health. Which physical health conditions are affected? To explore this, we construct a series of health measures from the individual health conditions reported in the data. Specifically, we construct five dummy variables for specific categories of conditions from the 15 listed in the data excluding the ‘other’ category. These are ‘skin/head/sight’ comprising health problems relating to skin, allergy, hearing and sight problems; ‘Cardio‐vascular disease’ comprising diabetes and heart/blood‐pressure problems; ‘respiratory’ comprising bronchial and asthmatic conditions; ‘musculoskeletal’ comprising arthritic/rheumatic conditions and ‘chronic’ comprising cancer, stroke and epilepsy (data for stroke and cancer is available from 2001 onwards).

We then estimate LPM of Equation (1) in which each of our health condition category dummy variables enters as the dependent variable. Results are shown in Table 3, Panel A. For the first four health condition categories, the coefficient on the house price variable is in each case negative and statistically significant at the 1% level of significance. The coefficients on the house price variables imply a 100% increase in house prices for skin/head/sight leads to a 5% reduction in likelihood (mean 0.23, SD 0.42); for cardio‐vascular disease, a 3% reduction (0.19, 0.39); for respiratory, a 4% reduction (0.12, 0.32) and for musculoskeletal, a 3% reduction. In the estimates for chronic health conditions (Column 5), the coefficient is also negative but only weakly statistically significant at the 5% level.

**Table 3:** Estimates of effects of house prices on specific health conditions, over 10‐year changes in house prices and health and on employment, hours of work and leisure

Panel A: fixed effects LPM estimates for types of health conditions					
	(1)	(2)	(3)	(4)	(5)
	Skin/head/sight	CVD	Respiratory	Musculo‐skeletal	Chronic
House prices	−0.0190** (0.00487)	−0.0131** (0.00437)	−0.0111** (0.00355)	−0.0130** (0.00433)	−0.0101* (0.00422)
R‐squared	0.025	0.068	0.018	0.030	0.024
No. obs.	104 992	105 170	104 992	104 992	105 170
No. groups	12 384	12 393	12 384	12 384	12 393
No. clusters	64	64	64	64	64
Panel B: LPM long‐difference estimates for effect of 10‐year house price changes on 10‐year changes in homeowner health					
	(1)	(2)	(3)	(4)	(5)
	No. conditions	SAH	Depression	GHQ	Death
∆ House prices	−0.141** (0.0325)	−0.0433* (0.0173)	−0.00552 (0.00588)	0.103 (0.0816)	−0.00414* (0.00176)
R‐squared	0.045	0.031	0.020	0.032	0.024
No. obs.	20031	20010	19990	19077	20031
Panel C: Fixed effects LPM estimates of effect of house prices on employment, hours of work, work capacity and leisure activities					
	(1)	(2)	(3)	(4)	
	Employment	Hours of work	Work capacity	Leisure activity	
House prices	−0.00777 (0.00536)	−0.335 (0.198)	−0.00702 (0.00447)	0.0143** (0.00370)	
R‐squared	0.069	0.062	0.067	0.620	
No. obs.	105 170	55 228	105 170	105 170	
No. groups	12 393	8171	12 393	12 393	
No. clusters	64	64	64	64	

Standard errors are in parentheses. Years 1993–2008 British Household Panel Survey homeowners sample comprises of head or household and partner/spouse. Models with county are presented by year dummies. Additional covariates included in model not shown in Panel A: age (in age brackets), relationship status dummies, educational achievement dummies and household composition dummies. Model in Panel B was estimated using 10‐year differences for sample from 1993–1998 to 2003–2008. Models with county are presented by year dummies. Additional covariates included in model (in 10‐year differences) not shown in table: local unemployment rate, dummy variables for whether the individual is employed, self‐employed, unemployed, annual income, age (in age brackets), relationship status dummies, educational achievement dummies and household composition dummies. Models with county are presented by year dummies. Panel C additional covariates included in model not shown in table: age (in age brackets), relationship status dummies, educational achievement dummies and household composition dummies. Cluster standard errors are in parentheses.

LPM, linear probability models; CVD, cardio‐vascular disease; SAH, Self‐assessed health; GHQ, General Health Questionnaire.

* *p* < 0.05,

** *p* < 0.01.

### 4.3 Long‐difference specifications

The results shown so far are based upon models with individual FE which exploit transitory changes in house prices and health. However, are these effects long lived? To explore this, we estimate long‐difference models in Equation (2). Results are shown in Table 3, Panel B. The coefficient on the 10‐year change house price variable is negative and statistically significant for both the number of health problems and SAH outcome variables. The coefficient value on the house price term in Column 1 is −0.141. Evaluated against the standard deviation of the dependent variable (1.15), this implies a 100% increase in house price over a 10‐year period which causes an 11% reduction in the number of health conditions over the sample period (mean 0.48). The coefficient value of −0.433 in Column 2 implies the effect on SAH is a 4.6% reduction in the SAH scale.

Column 5 of Table 3, Panel B presents coefficient estimates from the mortality model. The coefficient on the house price variable is negative, statistically significant at the 5% level and implies a 100% increase in house prices which reduces the probability of death by 0.4%; this is approximately one quarter of a standard deviation of the likelihood of death in our sample. Therefore, there is some evidence, conditional on estimation from a selected sample, that house price increases reduce the likelihood of death over longer periods of time.

### 4.4 Mechanisms and results for work and leisure

Why do house prices affect health? What are the ‘mechanisms’ by which house prices lead to changes in the health of individuals in our data? Unfortunately, the BHPS data contains limited information on health production. We have no information on nutritional diet and weight and little information on other health behaviours such as exercise and medical care.

We have examined the effect of house prices on risky health behaviours – smoking and drinking. Here, estimates indicate no statistically significant effect of house prices on these health behaviours. We have also explored the effects of house prices on use of private medical care. Results indicate house prices increase the likelihood of holding private healthcare coverage, but less than 5% of our sample holds private coverage, so this mechanism might only account for a very small fraction of the average effect we find in our estimates.

One potential mechanism by which house prices might affect health for which the BHPS provides extensive data is for labour market activity. House prices might have wealth effects on labour supply with individuals choosing to spend some of their housing wealth gains on increased leisure and less work. Alternatively, on the intensive margin, workers may respond to house price increases by reducing their hours of work. Reduced work hours lead to increases in sleep (Biddle and Hamermesh, 1990) which is linked to lower rates of obesity and related conditions such as musculoskeletal disorders (Sparks and Cooper, 1997; Spivey, 2010). A second channel could be through increased physical exercise and home food production (Petersen and Pedersen, 2004; Williamson and Pahor, 2010; Courtemanche, 2009).

Table 3, Panel C presents estimates from models in which we use our framework of Equation (1) to estimate models for labour market outcome variables. Columns 1 and 2 model labour supply: whether the individual is employed (Column 1) and the total number of hours worked per annum (Column 2). In Column 3, the dependent variable is a 1/0 dummy variable to indicate whether the individual's capacity to work is limited by their health. In Column 4, the dependent variable is a 1/0 dummy variable to indicate whether the individual participates in sport or exercise at least once per week.

Results show house price have no statistically significant effect on employment decisions but have a statistically significant effect on hours of work. The coefficient in Column 2 of Panel A is negative and statistically significant at the 1% level. The coefficient value of −0.335 implies a 100% increase in house prices reduces hours of work by on average 3.4% per annum. Estimates in Columns 3 and 4 show weak effects of house prices on work capacity but a statistically significant and positive effect on leisure activity. We suggest these results for the effects of house prices on labour supply decisions, and leisure may be one channel through which house prices affect health. However, more work is needed on this topic.

### 4.5 Non‐linear models and house price rises and falls

To further explore the relationship between house prices and health, we have estimated a variety of additional models. First, our main model assumes a linear relationship between house prices and health. Results from a model in which house prices enter as a quadratic function (Table A‐VI) show evidence for non‐linear effects. In the models for the number of health conditions and for SAH positive coefficients on the second order polynomial terms indicate diminishing marginal health gains from house price increases.

Second, we estimate Equation (5) that allows for asymmetric effects of house price rises and falls on individual health (Table A‐VII). Results from these models show asymmetric effects, with strong and statistically significant effects from house price booms (including effects on psychological health) but no statistically significant effects from house price busts. The more symmetric responses of SAH might be due to our definition of booms and busts as deviations from the long‐term mean.

## 5 Conclusion

We estimate the effect of wealth shocks on health using house prices as a source of large wealth gains and losses for home owning households. We find a positive and significant effect of house prices on health. A one standard deviation increase in house prices (which is close to a 100% increase in prices in our sample) leads to a decrease in the number of health conditions suffered by an individual of 6.6% of standard deviation and an improvement in SAH of approximately 4.3% of a standard deviation.

We show that our estimates are robust to alternative econometric models controlling for omitted local economic factors which might correlate with house prices and health plus reverse causality. There is no evidence for a purely psychological effect of house price changes, or that our estimates are confounded by home moving activity or self‐reporting bias. The health effects of house prices are not simply transitory but persist over 10‐year changes in our long‐difference estimates.

## Supporting information

Supporting info item

Supporting info item
