# Supplementary material for: Do Wealth Shocks Affect Health? New Evidence from the Housing Boom
Source: Health Econ. 2016 Nov 9;25(Suppl Suppl 2):57–69. doi: 10.1002/hec.3431 (PMC5111776; doi:10.1002/hec.3431)
Supplement: Supplementary file 1 — Supporting info item [file HEC-25-57-s001.docx]

Online Appendix Accompanying

**‘Do Wealth Shocks Affect Health?
New Evidence from the Housing Boom’**

This online appendix accompanies figures and tables included in the main text. Tables in this appendix are prefixed A-, hence A-I, A-II and so on, and are referenced in the main text.

Table A-I. Fixed Effects LPM Estimates of Effect of House Prices on Homeowner Health – Robustness Specifications

Table A-II. Fixed Effects Estimates of Effect of House Prices on Homeowner Health using Predicted House Prices and GMM

Table A-III. Fixed Effects LPM Estimates of Effect of House Prices on Homeowner Health Including Lagged House Prices

Table A-IV. Fixed Effects LPM Estimates of Effect of House Prices on Homeowner Health Excluding Movers and with Balanced Sample of Homeowners

Table A-V. Correlated Random Effects Linear and Non-Linear Models of Effects of House Prices on Homeowner Health

Table A-VI. Fixed Effects LPM Estimates of Effect of House Prices on Homeowner Health Including Polynomial Specification

Table A-VII. Fixed Effects LPM Estimates of Effect of House Prices with Booms and Busts

Table A-I. Fixed Effects LPM Estimates of Effect of House Prices on Homeowner Health – Robustness Specifications

| Panel A: Bootstrap Standard Errors | | | | |
| --- | --- | --- | --- | --- |
|  | (1) | (2) | (3) | (4) |
|  | No. conditions | SAH | Depression | GHQ |
| House Prices | -0.0865^**^ (0.0123) | -0.0368^**^ (0.00995) | -0.00709 (0.00378) | -0.0224 (0.0389) |
| County Unemployment | -0.0120 (0.00633) | -0.000265 (0.00460) | -0.00205 (0.00184) | 0.0110 (0.0164) |
| Annual Income | -0.00398 (0.00728) | -0.00481 (0.00594) | 0.000513 (0.00220) | 0.0229 (0.0203) |
| Employed | -0.0974^**^ (0.0142) | -0.110^**^ (0.0118) | -0.0119^*^ (0.00501) | -0.341^**^ (0.0446) |
| Self-employed | -0.118^**^ (0.0206) | -0.135^**^ (0.0169) | -0.00872 (0.00639) | -0.304^**^ (0.0653) |
| Unemployed | -0.0783^**^ (0.0301) | -0.0727^**^ (0.0238) | 0.00713 (0.00973) | 0.847^**^ (0.106) |
| R-squared | 0.064 | 0.017 | 0.003 | 0.009 |
| No. obs. | 105170 | 97177 | 104992 | 101325 |
| No. groups | 12393 | 12107 | 12384 | 12090 |
| No. clusters | 12393 | 12107 | 12384 | 12090 |
| Panel B: County Time Trends | | | | |
|  | (1) | (2) | (3) | (4) |
|  | No. conditions | SAH | Depression | GHQ |
| House Prices | -0.0851^**^ (0.0139) | -0.0366^**^ (0.00966) | -0.00607 (0.00395) | -0.0134 (0.0453) |
| County Unemployment | -0.0147 (0.00989) | 0.00265 (0.00614) | 0.000495 (0.00273) | 0.0227 (0.0232) |
| Annual Income | -0.00552 (0.00804) | -0.00537 (0.00590) | 0.000684 (0.00226) | 0.0195 (0.0252) |
| Employed = 1 | -0.100^**^ (0.0158) | -0.111^**^ (0.00901) | -0.0122^**^ (0.00355) | -0.346^**^ (0.0479) |
| Self-employed = 1 | -0.121^**^ (0.0238) | -0.134^**^ (0.0163) | -0.00918 (0.00599) | -0.312^**^ (0.0639) |
| Unemployed = 1 | -0.0783^*^ (0.0321) | -0.0712^**^ (0.0210) | 0.00664 (0.00888) | 0.843^**^ (0.102) |
| R-squared | 0.067 | 0.019 | 0.005 | 0.011 |
| No. obs. | 105170 | 97177 | 104992 | 101325 |
| No. groups | 12393 | 12107 | 12384 | 12090 |
| No. clusters | 64 | 64 | 64 | 64 |

Standard errors in parentheses. Note 1993-2008 BHPS homeowners sample comprising head or household and partner/spouse. Additional covariates included in model not shown in table: age (in age brackets), relationship status dummies, educational achievement dummies, household composition dummies. Cluster std. errors in ().

^*^ *p* < 0.05, ^**^ *p* < 0.01

Table A-II. FE Estimates of Effect of House Prices on Homeowner Health using Predicted House Prices and GMM

| Panel A: Predicted House Prices Using County Level Index | | | | |
| --- | --- | --- | --- | --- |
|  | (1) | (2) | (3) | (4) |
|  | No. conditions | SAH | Depression | GHQ |
| Predicted house prices | -0.188^**^ (0.0312) | -0.0292 (0.0201) | 0.0141 (0.00774) | 0.166 (0.0861) |
| R-squared | 0.067 | 0.019 | 0.005 | 0.011 |
| No. obs. | 105098 | 97108 | 104920 | 101261 |
| No. groups | 12392 | 12106 | 12383 | 12089 |
| No. clusters | 64 | 64 | 64 | 64 |
| Panel B: GMM (Order – 3) Estimates | | | | |
|  | (1) | (2) | (3) | (4) |
|  | No. conditions | SAH | Depression | GHQ |
| House prices | -0.276^**^ (0.0672) | -0.0899 (0.0548) | 0.000974 (0.0220) | 0.253 (0.208) |
| R-squared | 0.044 | 0.015 | 0.006 | 0.011 |
| No. obs. | 67916 | 63493 | 67825 | 65850 |
| No. groups | 8503 | 8471 | 8498 | 8335 |

Standard errors in parentheses. Note 1993-2008 BHPS homeowners sample comprising head or household and partner/spouse. Models with county time trends. House value predicted from fixed effects LPM including county house prices, house type, number of rooms, original ownership status; GMM model used lagged house prices of order three and beyond. Additional covariates included in model not shown in table: age (in age brackets), relationship status dummies, educational achievement dummies, household composition dummies.

^*^ *p* < 0.05, ^**^ *p* < 0.01

Table A-III. Fixed Effects LPM Estimates of Effect of House Prices on Homeowner Health Including Lagged House Prices

|  | (1) | (2) | (3) | (4) |
| --- | --- | --- | --- | --- |
|  | No. conditions | SAH | Depression | GHQ |
| House prices | -0.0417^*^ (0.0158) | -.0324^*^ (0.0138) | -0.00653 (0.00568) | 0.000942 (0.0611) |
| House prices t-1 | -0.00755 (0.0181) | -0.00113 (0.0145) | 0.000590 0.00547) | 0.0269 (0.0477) |
| House prices t-2 | -0.0215 (0.0190) | -0.00176 (0.0138) | -0.00130 (0.00525) | 0.000373 (0.0419) |
| House prices t-3 | -0.0234 (0.0150) | -0.0103 (0.0162) | 0.0101 (0.00530) | 0.154^**^ (0.0458) |
| R-squared | 0.060 | 0.026 | 0.016 | 0.022 |
| No. obs. | 67605 | 63252 | 67526 | 65634 |
| No. groups | 9116 | 9086 | 9112 | 8966 |
| No. clusters | 64 | 64 | 64 | 64 |

Standard errors in parentheses. Note 1993-2008 BHPS homeowners sample comprising head or household and partner/spouse. Models with county by year dummies. Additional covariates included in model not shown in table: age (in age brackets), relationship status dummies, educational achievement dummies, household composition dummies. Cluster std. errors in (). ^*^ *p* < 0.05, ^**^ *p* < 0.01

Table A-IV. Fixed Effects LPM Estimates of Effect of House Prices on Homeowner Health Excluding Movers and with Balanced Sample of Homeowners

| Panel A: Excluding Movers | | | | |
| --- | --- | --- | --- | --- |
|  | (1) | (2) | (3) | (4) |
|  | No. conditions | SAH | Depression | GHQ |
| House Prices | -0.0761^**^ (0.0145) | -0.0320^**^ (0.0112) | -0.00658 (0.00477) | -0.0183 (0.0538) |
| County Unemployment | 0.00480 (0.0190) | 0.0100 (0.00997) | 0.00260 (0.00696) | 0.0519 (0.0452) |
| Annual Income | -0.00209 (0.00861) | -0.00452 (0.00649) | 0.000686 (0.00233) | 0.0139 (0.0272) |
| Employed = 1 | -0.105^**^ (0.0164) | -0.115^**^ (0.00968) | -0.0129^**^ (0.00371) | -0.340^**^ (0.0517) |
| Self-employed = 1 | -0.121^**^ (0.0251) | -0.142^**^ (0.0165) | -0.0106 (0.00586) | -0.341^**^ (0.0674) |
| Unemployed = 1 | -0.0712^*^ (0.0318) | -0.0648^**^ (0.0210) | 0.0111 (0.0101) | 0.885^**^ (0.109) |
| R-squared | 0.079 | 0.029 | 0.014 | 0.021 |
| No. obs. | 99099 | 91489 | 98933 | 95446 |
| No. groups | 12002 | 11713 | 11993 | 11726 |
| No. clusters | 64 | 64 | 64 | 64 |
| Panel B: Balanced Panel Home Owners | | | | |
|  | (1) | (2) | (3) | (4) |
|  | No. conditions | SAH | Depression | GHQ |
| House Prices | -0.0819^**^ (0.0168) | -0.0417^**^ (0.0127) | -0.00752 (0.00493) | -0.0174 (0.0523) |
| County Unemployment | 0.00723 (0.0210) | 0.0122 (0.0118) | 0.00196 (0.00717) | 0.0505 (0.0556) |
| Annual Income | -0.00330 (0.00930) | -0.0131 (0.00738) | 0.00125 (0.00266) | 0.0239 (0.0310) |
| Employed = 1 | -0.108^**^ (0.0158) | -0.113^**^ (0.0122) | -0.0137^**^ (0.00424) | -0.296^**^ (0.0559) |
| Self-employed = 1 | -0.128^**^ (0.0258) | -0.139^**^ (0.0224) | -0.00887 (0.00716) | -0.324^**^ (0.0749) |
| Unemployed = 1 | -0.0983^**^ (0.0328) | -0.0652^*^ (0.0268) | -0.000189 (0.00899) | 0.928^**^ (0.111) |
| R-squared | 0.085 | 0.031 | 0.016 | 0.024 |
| No. obs. | 82517 | 76432 | 82384 | 79460 |
| No. groups | 8929 | 8708 | 8922 | 8680 |
| No. clusters | 64 | 64 | 64 | 64 |

Standard errors in parentheses. Note 1993-2008 BHPS homeowners sample comprising head or household and partner/spouse. Models with county by year dummies. Additional covariates included in model not shown in table: age (in age brackets), relationship status dummies, educational achievement dummies, household composition dummies. Cluster std. errors in (). ^*^ *p* < 0.05, ^**^ *p* < 0.01

Table A-V. Correlated Random Effects Linear and Non-Linear Models of Effects of House Prices on Homeowner Health

| Panel A: Mundlak Correlated Random Effects | | | | |
| --- | --- | --- | --- | --- |
|  | (1) | (2) | (3) | (4) |
|  | No. conditions | SAH | Depression | GHQ |
| House Prices | -0.0622^**^ (0.0105) | -0.0278^**^ (0.00821) | -0.00753^*^ (0.00343) | -0.00284 (0.0307) |
| County Unemployment | 0.00930 (0.00911) | -0.000314 (0.00316) | 0.00331 (0.00280) | 0.00682 (0.0117) |
| Annual Income | -0.00527 (0.00586) | -0.00409 (0.00494) | 0.000588 (0.00194) | 0.0239 (0.0185) |
| Employed = 1 | -0.104^**^ (0.0107) | -0.113^**^ (0.00892) | -0.0118^**^ (0.00353) | -0.340^**^ (0.0334) |
| Self-employed = 1 | -0.127^**^ (0.0165) | -0.137^**^ (0.0138) | -0.00882 (0.00548) | -0.301^**^ (0.0520) |
| Unemployed = 1 | -0.0813^**^ (0.0241) | -0.0753^**^ (0.0201) | 0.00691 (0.00798) | 0.839^**^ (0.0759) |
| No. obs. | 105170 | 97177 | 104992 | 101325 |
| No. groups | 12393 | 12107 | 12384 | 12090 |
| Panel B: Non-Linear Estimators (Fixed Effects Poisson, Ordered Probit) | | | | |
|  | (1) | (2) | (3) | (4) |
|  | No. conditions | SAH | Depression | GHQ |
| House Prices | -0.0169^**^ (0.00132) | -0.0533^**^ (0.0164) | -0.0861^*^ (0.0416) | -0.0119 (0.0160) |
| County Unemployment | 0.00817 (0.0111) | -0.00176 (0.00629) | -0.0155 (0.0116) | 0.00879 (0.00615) |
| Annual Income | -0.000755 (0.00748) | -0.00718 (0.00984) | 0.00812 (0.0177) | 0.00837 (0.00967) |
| Employed = 1 | -0.0728^**^ (0.0136) | -0.208^**^ (0.0177) | -0.0957^**^ (0.0307) | -0.168^**^ (0.0172) |
| Self-employed =1 | -0.0834^**^ (0.0225) | -0.260^**^ (0.0275) | -0.0551 (0.0528) | -0.165^**^ (0.0273) |
| Unemployed = 1 | -0.0465 (0.0311) | -0.140^**^ (0.0396) | 0.0699 (0.0668) | 0.347^**^ (0.0372) |
| No. obs. | 105170 | 97177 | 104939 | 101325 |
| No. groups | 12393 | 12107 | 12381 | 12090 |

Coefficients displayed and Standard errors in parentheses. Note 1993-2008 BHPS homeowners sample comprising head or household and partner/spouse. Models with county by year dummies. Additional covariates included in model not shown in table: age (in age brackets), relationship status dummies, educational achievement dummies, household composition dummies and mean of time varying covariates. Models (1-4) in Panel A are Mundlak correlated random effect models; In Panel B Models (1, 4) are Poisson model; Model (2) is an ordered probit models; Model (3) is probit model. ^*^ *p* < 0.05, ^**^ *p* < 0.01

Table A-VI. Fixed Effects LPM Estimates of Effect of House Prices on Homeowner Health Including Polynomial Specification

|  | (1) | (2) | (3) | (4) |
| --- | --- | --- | --- | --- |
|  | No. conditions | SAH | Depression | GHQ |
| House prices | -0.00381^**^ (0.000722) | -0.00198^**^ (0.000558) | -0.00000117 (0.000238) | 0.000883 (0.00265) |
| House prices Squared | 0.0756^**^ (0.0231) | 0.0295^*^ (0.0113) | -0.000921 (0.00436) | -0.0333 (0.0507) |
| R-squared | 0.076 | 0.028 | 0.013 | 0.020 |
| No. obs. | 105170 | 97177 | 104992 | 101325 |
| No. groups | 12393 | 12107 | 12384 | 12090 |
| No. clusters | 64 | 64 | 64 | 64 |

Standard errors in parentheses. Note 1993-2008 BHPS homeowners sample comprising head or household and partner/spouse. Models with county by year dummies. Additional covariates included in model not shown in table: age (in age brackets), relationship status dummies, educational achievement dummies, household composition dummies. Cluster std. errors in (). ^*^ *p* < 0.05, ^**^ *p* < 0.01

Table A-VII. Fixed Effects LPM Estimates of Effect of House Prices with Booms and Busts

|  | (1) | (2) | (3) | (4) |
| --- | --- | --- | --- | --- |
|  | No. conditions | SAH | Depression | GHQ |
| House Price Booms | -0.0939^**^ (0.0265) | -0.127^**^ (0.0157) | -0.0254^**^ (0.00734) | -0.160^**^ (0.0510) |
| House Price Busts | -0.0618 (0.0408) | -0.0997^**^ (0.0188) | -0.0108 (0.00699) | -0.0263 (0.0561) |
| County Unemployment | 0.00519 (0.00743) | -0.00684 (0.00426) | 0.00107 (0.00157) | -0.0481^**^ (0.0126) |
| Yearly Income | -0.0374^**^ (0.0121) | -0.0589^**^ (0.00890) | -0.0126^**^ (0.00336) | -0.0789^*^ (0.0353) |
| Employed = 1 | -0.547^**^ (0.0344) | -0.378^**^ (0.0218) | -0.0832^**^ (0.00864) | -0.683^**^ (0.0598) |
| Self-employed = 1 | -0.582^**^ (0.0379) | -0.399^**^ (0.0255) | -0.104^**^ (0.0101) | -0.780^**^ (0.0733) |
| Unemployed = 1 | -0.408^**^ (0.0483) | -0.173^**^ (0.0285) | -0.0470^**^ (0.0144) | 0.734^**^ (0.127) |
| R-squared | 0.168 | 0.078 | 0.017 | 0.019 |
| No. obs. | 105170 | 97177 | 104992 | 101325 |
| No. clusters | 64 | 64 | 64 | 64 |

Standard errors in parentheses. Note 1993-2008 BHPS homeowners sample comprising head or household and partner/spouse. Models with county by year dummies. Additional covariates included in model not shown in table: age (in age brackets), relationship status dummies, educational achievement dummies, household composition dummies. Cluster std. errors in (). ^*^ *p* < 0.05, ^**^ *p* < 0.01
