# Supplementary material for: Do Wealth Shocks Affect Health? New Evidence from the Housing Boom
Source: Health Econ. 2016 Nov 9;25(Suppl Suppl 2):57–69. doi: 10.1002/hec.3431 (PMC5111776; doi:10.1002/hec.3431)
Supplement: Supplementary file 2 — Supporting info item [file HEC-25-57-s002.docx]

**Do Wealth Shocks Affect Health?
New Evidence from the Housing Boom**

Author response to reviewer comments – revision 2

We would like to thank the editor and referee for their helpful comments on the revised version of our paper. Below we address the remaining points raised by Reviewer 1. We include the reviewer’s comments in italics and our responses in plain type:

**Reviewer: 1**

*This revised version of the manuscript is considerably improved over the previous draft. However, in my view, a number of specific changes would improve it further. These are detailed below.*

*1.	The writing throughout, but particularly in the first couple of sections, still needs to be tightened up and carefully edited.*

We have taken time to revise the text throughout, aiming for clarity and a concise style. The introduction in the new version is shorter and we have removed some repetitive content. Overall the paper in now 25 pages in length, including references, figures and tables.

*2.	Although the tables are better, as mentioned in my previous comments, we do not need to see all of the coefficients for the supplementary coefficients each time. My suggestion would be to show them once, in Table II, and then only show the home price, or change in home price, coefficients for the remaining coefficients. Once this is done, Tables III through V could be combined into a single remaining table (but see my comment on the material in Table 5 below).*

We have combined the results from Tables III – V (excluding the top panel of Table V which the referee asks us to remove at point 4 below) into a new version of Table III comprising 3 panels of results. We have updated the cross-references in the text accordingly.

*3.	I’m still troubled by the use of linear models for self-assessed health and the GHQ scores. At a minimum, estimate models for excellent/very good vs. good/fair/poor or poor/fair vs. good/very good/excellent self-assessed health and for ≥1 versus 0 GHQ scores. If consistent with the other models, these results could just be reported in the sections on robustness checks, rather than being shown in tables.*

In Table A-V of the paper on-line Appendix, we had reported non-linear models of the number of health conditions (Poisson model); Self-Assessed health and GHQ (ordered probit models); and Depression (probit model). The results are qualitatively similar to the linear models.

We also follow the Referee’s suggestion and we estimate binary probit models of SAH (equal to one if self-assessed health is fair, poor or very poor and 0 otherwise) and GHQ (equal to one if GHQ is greater or equal than one). The results (reported below) are qualitatively similar to the ordered probit models and the linear models reported in the paper.

Table R1 Binary Probit models of the Effects of House Prices on Homeowner self-assessed health and GHQ

|  | (1) | (2) |
| --- | --- | --- |
|  | SAH | GHQ |
| House prices | -0.051^*^ (0.023) | -0.029 (0.019) |
| County Unemployment | 0.016 (0.009) | 0.013 (0.007) |
| Annual Income | -0.015 (0.014) | 0.005 (0.011) |
| Employed=1 | -0.236^**^ (0.038) | -0.138^**^ (0.020) |
| Self-employed=1 | -0.297^**^ (0.054) | -0.153^**^ (0.032) |
| Unemployed=1 | -0.096 (0.054) | 0.294^**^ (0.046) |
| No. obs. | 97,169 | 105,170 |
| No. groups | 12,107 | 12,393 |

Coefficients displayed and Standard errors in parentheses. Note 1993-2008 BHPS homeowners sample comprising head or household and partner/spouse. Models with county and year dummies. SAH equals to one if self-assessed health is fair, poor or very poor and 0 otherwise. GHQ equals to one if GHQ is greater or equal than one. Additional covariates included in model not shown in table: age (in age brackets), relationship status dummies, educational achievement dummies, household composition dummies and mean of time varying covariates. Models (1-2) in Panel A are Mundlak correlated random effect models.

*4.	The estimates in the top panel of Table V make little. For instance, including unemployment status in a model where the dependent variable is whether or not the individual is employed is odd and confusing at best. Only show estimates from the bottom panel.*

We have omitted the top panel of Table V from the new version, and have combined the tables as suggested above at point 3. We have updated the text accordingly.
